# AZO Nanoparticles-Decorated CNTs for UV Light Sensing: A Structural, Chemical, and Electro-Optical Investigation

**DOI:** 10.3390/nano13010215

**Published:** 2023-01-03

**Authors:** Simona Filice, Stefano Boscarino, Mario Scuderi, Sebania Libertino, Clelia Galati, Antonio Terrasi, Silvia Scalese

**Affiliations:** 1 Consiglio Nazionale delle Ricerche—Istituto per la Microelettronica e Microsistemi (CNR-IMM), Ottava Strada n.5, I-95121 Catania, Italy; 2 Dipartimento di Fisica e Astronomia, Università degli Studi di Catania, Via S. Sofia 64, I-95123 Catania, Italy; 3 Consiglio Nazionale delle Ricerche—Istituto per la Microelettronica e Microsistemi (CNR-IMM), sede di Catania—Università, Via S. Sofia 64, I-95123 Catania, Italy; 4 STMicroelectronics, Stradale Primosole 5, I-95121 Catania, Italy

**Keywords:** MWCNTs, ZnO, AZO, nanoparticles, UV sensor

## Abstract

Nanocomposites formed by aluminum-doped zinc oxide nanoparticles (AZO–NP) and multiwall carbon nanotubes (CNT) are proposed here as a promising material for UV light sensing applications, with the great advantage of operating in air, at room temperature, and at low voltage. Nanocomposite layers were prepared with different AZO:CNT weight ratios by a simple methodology at room temperature. They were characterized by means of UV–Vis spectroscopy, scanning and transmission electron microscopies (SEM and TEM), and X-ray photoelectron spectroscopy (XPS). The interaction between the two nanomaterials was demonstrated by comparing the properties of the nanocomposite with the ones shown by the AZO–NPs. Dense AZO–CNT nanocomposite layers were deposited between two metal electrodes on a SiO_2_/Si substrate, and the electrical properties were investigated in dark condition and under UV light irradiation. The electrical response to the UV light was a sudden current increase that reduced when the light was switched off. Several UV on/off cycles were performed, showing good repeatability and stability of the response. The mechanisms involved in the electrical response are discussed and compared to the ones previously reported for ZnO–CNT nanocomposites.

## 1. Introduction

Today, novel UV sensors with high sensitivity operating at room temperature, with low power consume and with high thermal and electric stability are highly required. 

An attractive method to achieve this goal is to couple metal oxide semiconductors such as ZnO or AZO, in the form of nanoparticles, with carbon nanotubes, leading to the formation of a hybrid system mixing the properties of both components. Metal oxides and their interaction with light are the subject of several studies in different fields, e.g., photocatalysis, antimicrobial coatings, and sensing [1,2,3,4,5,6]. Zinc oxide (ZnO) and aluminum-doped zinc oxide (AZO), in particular, have gained a lot of attention in sensing applications to detect gases [7,8,9,10,11], UV light [12,13,14,15], and humidity [16]. ZnO is an important II–VI group semiconductor with a wide direct bandgap of 3.3 eV, depending on the type of synthesis/deposition method [17,18]. Due to the wide bandgap, ZnO is suitable to be used as highly sensitive material in UV photodetector sensing layers. The ability of photocurrent generation depends on the wavelength of the illumination light, and in this case, the highest photosensitivity can be achieved in the wavelength range between 340 and 400 nm. For electrical transduction sensors, detection is performed by measuring electrical current changes occurring during exposure to light irradiation, gases, and other species; therefore, the high electrical resistivity of ZnO at room temperature can be a hindrance. Indeed, it is often required to operate at high temperatures (200–500 °C) in order to increase the measured current and get sufficient sensitivity. Reducing the operating temperature to room temperature would allow for a reduction in the energy consumption during sensors’ operation and avoid gas explosion in the presence of flammable and explosive gases. The low electrical conductivity and the optical transparency in the visible region of ZnO films or nanostructures can be improved by doping, i.e., by substituting Zn^2+^ cations with group III elements (B, Al, Ga, or In) or group IV elements (Si, Ti, or Sn). In particular, Al is one of the most suitable dopants of ZnO wurtzite crystals, and the optical and electrical properties of ZnO can be modified by controlling the doping level, allowing for an enhancement of the sensing response.

A useful approach to further increase the electrical response of metal oxide layers for sensing applications is the preparation of composites formed by metal oxides and conductive materials (e.g., carbon-based structures), the first ones playing the role of sensitive material, the latter playing the role of electrical transducers. In previous work, we have reported the preparation of a nanocomposite layer formed by ZnO nanoparticles and CNTs [19], showing good sensitivity to UV light and a noteworthy electrical response (up to 9.8% increase of electrical resistance under UV irradiation, normalized to the resistance value in the dark for the investigated conditions). It was shown that the electrical response of the nanocomposite material was dependent on the ZnO:CNT ratio and on the total irradiance, suggesting that these materials could provide not only qualitative information (UV light on–off condition), but also quantitative information about the source irradiance to which they are exposed. As concerns the comparison with similar sensing devices, the proposed ones operate at 2 mV instead of the typical 10 V [14,20,21], and within a very small active area (10^–3^ mm^2^ instead of typical values between 0.1–1 cm^2^). 

Al doping introduces into the ZnO nanoparticle an extra negative charge that may affect the sensing response to light irradiation. This holds true also for AZO–CNT nanocomposites, where charge transfer between the two nanomaterials is possible. Ahmad et al. reported the preparation and use of Al-doped ZnO–CNTs and Al-doped ZnO/graphene in photocatalysis: they prepared the photocatalysts by the solvothermal route [22].. Ahn et al. investigated the NO gas sensing characteristics of layered composites fabricated by coaxial coating of single-walled CNTs with a thin layer of 1 wt% Al-doped ZnO using RF magnetron sputtering deposition [23]. These approaches require many steps and a high operating temperature.

The AZO–CNT preparation methodology here proposed allowed us to reduce costs and to operate in normal environmental conditions, which could be extended to a larger area and low-cost substrates. The electrical properties of this material have been investigated in dark conditions and under UV light irradiation (200–400 nm wavelength range): as the AZO–CNT layers are exposed to UV light, the current measured through the layer increases and returns to lower values when light is switched off, showing an electrical response opposite to the one observed for ZnO–CNT. This can be explained considering the extra free carriers present in the AZO conduction band due to Al doping, and other mechanisms such as the irradiation process and the charge transfer between AZO and CNTs.

## 2. Materials and Methods

Multiwalled carbon nanotubes (CNT) with an average diameter/length of 9.5 nm/1.5 μm, respectively, and carboxylic acid functionalization were purchased from Sigma–Aldrich S.r.l. (Milan, Italy). The –COOH functional groups present on the carbon nanotubes allow for better dispersion in solution. 

AZO nanoparticles have a nominal size of about 15 nm and are suspended in a mixture of low- and high-boiling alcohols of approximately 10 cP viscosity; the solid content was 2.5 wt.%. For comparison, a 2.5 wt.% dispersion of crystalline ZnO (hexagonal wurtzite) nanoparticles in isopropanol (IPA) was used. Both AZO and ZnO nanoparticles were supplied by Avantama AG (Stafa, Switzerland). The average diameter of the ZnO nanoparticles wes about 15 nm.

AZO–CNT solutions were prepared by dispersing metal oxide nanoparticles and CNTs in isopropanol separately; then they were sonicated for 1 h at room temperature. Two CNT concentrations (1 μg/mL and 5 μg/mL) and two AZO concentrations (33 μg/mL and 21 μg/mL) were used for the experiments. Lastly, the solutions were mixed together at different AZO:CNT weight ratios and sonicated at room temperature again. 

The AZO-decorated CNTs were characterized by UV–Vis spectroscopy, using a UV/Vis Cary 50 spectrophotometer by AGILENT (Santa Clara, CA, USA) in a wavelength range between 200 and 800 nm. Morphological and structural characterization was performed by scanning electron microscopy (SEM), using a field emission scanning electron microscope (Supra35 FE-SEM by Zeiss, Oberkochen, Germany). Transmission electron microscopy (TEM) was used for a nanoscale structural characterization and was performed in a probe Cs-corrected JEM-ARM200F by JEOL (Akishima, Tokyo), equipped with a cold FEG operated at a primary beam energy of 200 keV in both conventional TEM (CTEM) and in scanning TEM (STEM) modes, and with a GIF Quantum ER energy filter by Gatan Inc. (Pleasanton, CA, USA) used for electron energy loss spectroscopy (EELS) measurements.

Chemical analysis was performed by X-ray photoelectron spectroscopy (XPS), using Al K_α_ radiation (1486.5 eV) as the photon source and a VG CLAM4 multichanneltron detector hemispherical analyzer. A pass energy of 20 eV was used for all scans, giving an overall resolution of 1 eV.

Metal oxide nanoparticle-decorated CNTs were deposited by dielectrophoresis between two metal electrodes obtained on a Si substrate, following a procedure reported in previous works [19,20,21,22,23,24]. In particular, a silicon dioxide (SiO_2_) layer was thermally grown on a silicon substrate, and two metal electrodes (Pt 100 nm/Ti 20 nm) were defined by optical lithography and lift-off process [24], with a distance between the electrodes of 4 μm and a width of 500 μm. Electrical measurements on the AZO–CNT layers were performed by applying a voltage of 2 mV between the electrodes, both in the dark and under UV irradiation, and measuring the current flowing through the layer, using a Keithley 6430 Sub-Femtoamp Remote Sourcemeter (by Keithley Instruments—Tektronix Inc., Beaverton, OR, USA). 

UV irradiation was performed using an ASBN-D130 deuterium light source by Spectral Products (Putnam, CT, USA). The light source has been previously characterized by a fiber-optic spectrometer (AvaSpec-ULS2048L). The spectrum is reported in Figure S1 of the supplementary materials.

## 3. Results

### 3.1. Optical Characterization

**Figure 1:**
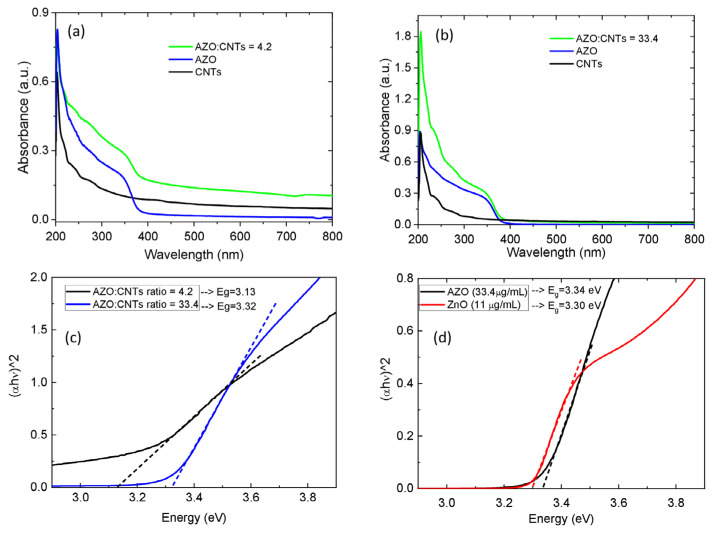
UV–Vis absorbance spectra of AZO–CNT solutions for (**a**) AZO:CNT weight ratio = 4.2 and (**b**) 33.4 spectra for AZO–NPs and CNTs solutions are added as comparison. In (**c**,**d**) Tauc plots for AZO–CNT composites and for AZO and ZnO nanoparticles, respectively, obtained considering a direct allowed transition (*n* = 2).

The AZO nanoparticles (AZO–NPs) and the AZO–NP-decorated CNTs dispersed in IPA were characterized by UV–Vis spectroscopy. The results are reported in Figure 1. We plotted the absorbance spectra of AZO–CNTs for two different weight ratios: AZO:CNT = 4.2, AZO:CNT = 33.4, and for AZO–NPs and CNTs separately (Figure 1a,b). From absorbance spectra, it is possible to extract the optical bandgap by using Tauc’s equation [18]:

(1) $${\left(\alpha hv\right)}^{n}=B\;\left(hv-E_{gap}\right)$$

where *h* is Planck’s constant; *ν* is the photon frequency; and α is the absorption coefficient. The corresponding Tauc plots for AZO–CNTs composites, AZO, and ZnO nanoparticles are reported in Figure 1c,d, respectively. We extrapolated the *E_gap_* from the energy axis intercept of the line fitted on the linear portion of the Tauc plot by considering a direct allowed transition (*n* = 2), as it is known for AZO and ZnO. The optical bandgap value for each solution is obtained by the intercept of the linear fit (linear portion of the plotted curves) with the hν axis. In particular, for AZO–NPs, the bandgap is found to be 3.32 eV, similar to the 3.30 eV found for ZnO–NPs, and reduces for AZO–NP-decorated CNTs as a function of the AZO:CNT weight ratio: almost no change is observed for AZO:CNT = 33.4, while it decreases to 3.13 eV for AZO:CNT = 4.2. Therefore, an interaction between AZO–NPs and CNTs is supposed to take place, but it is visible in the absorbance spectra only when most of the AZO–NPs are bound to CNTs, since spectra are given by a combination of both free AZO–NPs and bound AZO–NP contributions. For the lowest AZO:CNT weight ratio, we are confident that all (or most of) the AZO–NPs are interacting with CNTs. We will show below, in the electron microscopy analysis and the relative absorbance spectra, that they are mainly composed of the contribution of AZO–NPs bound to CNTs. On the contrary, for the highest AZO:CNT weight ratio, many AZO–NPs are free and their contribution to the optical signal is predominant. Indeed, a similar behavior was also observed for ZnO–NPs and ZnO-decorated CNTs in a previous work [19]: for ZnO-decorated CNTs (for an optimized ratio), the optical bandgap was 0.24 eV lower than for ZnO nanoparticles.

### 3.2. Morphological/Structural Characterization 

In order to investigate the morphology and the structural properties of the samples, we performed SEM and TEM analysis. For an AZO:CNT weight ratio of 4.2, we found the best condition to have most of the particles bound to CNTs, minimizing the free particle amount in solution. 

**Figure 2:**
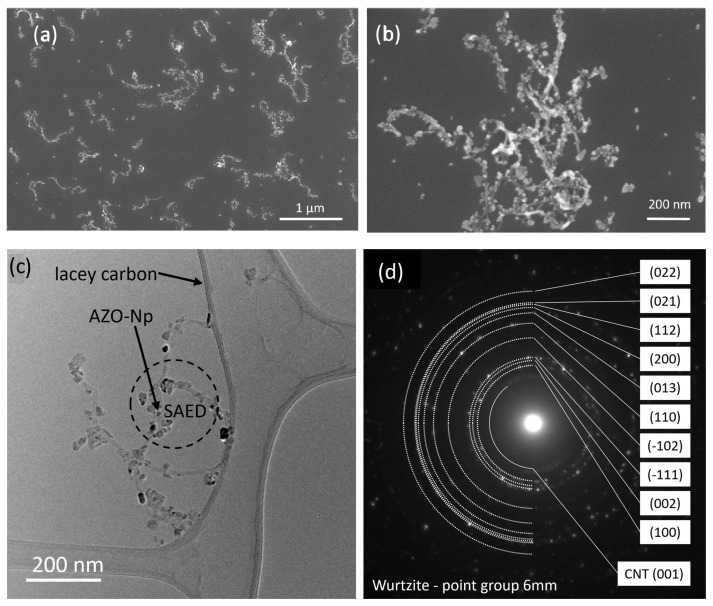
Lower (**a**) and higher (**b**) magnification SEM images of AZO-decorated CNTs, with an AZO:CNT weight ratio of 4.2; (**c**) TEM bright field image of AZO-decorated CNTs; (**d**) SAED pattern of the region indicated in (**c**).

In Figure 2a,b, we report SEM images at lower and higher magnification, respectively, showing that the CNTs are homogeneously decorated by AZO–NPs. A more detailed structural characterization was carried out by TEM analysis, as reported in Figure 2c,d. Figure 2c is a bright-field TEM image of some AZO-decorated CNTs lying on the surface of the thin support film of a lacy carbon TEM grid. The dashed circle indicates the region used to acquire the selected area electron diffraction (SAED) of Figure 2d that shows the pattern of a wurtzite-like structure (point group 6 mm) for the AZO–NPs; in addition, the (001)s at 3.5 Å of CNTs are also recognized. The interplanar distances of AZO–NPs are very close to that of ZnO, pointing out the little amount of aluminum on the AZO–NPs.

**Figure 3:**
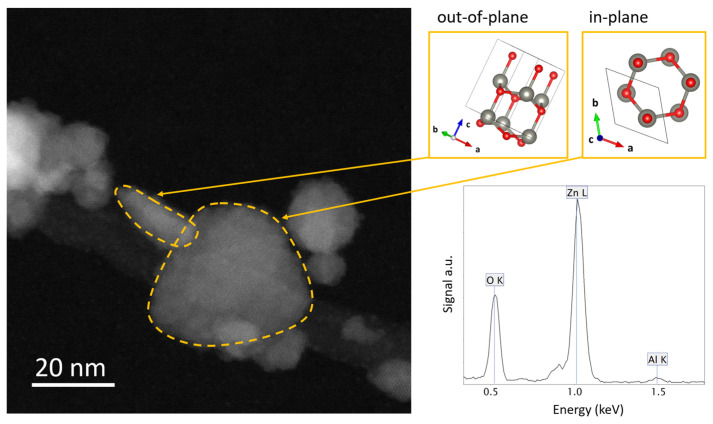
STEM Z-contrast image of an AZO-decorated CNT portion showing AZO–NPs with orthogonal orientations indicated by the atomic models on the upper-right panel. On the bottom right panel, an EDX spectrum of the AZO–NPs.

The AZO–NPs possess more likely a flattened disk shape, and the contact between the AZO–NPs and the CNTs occurs mainly through the disk face, that is, a (001) plane, as in the case of ZnO–NPs bound to CNTs, reported in our previous work [19]. In the STEM Z-contrast image of Figure 3, two AZO–NPs with different orientations are shown with dotted yellow lines. The first (out-of-plane view of the disk) shows the elongated shape of the disk and the contact point between the AZO–NP and the CNT, while the second (in-plane view) shows the disk face. The latter mostly exhibits a triangular/hexagonal shape emerging from the symmetry of the wurtzite structure on the (001) planes. Energy-dispersive X-ray (EDX) spectroscopy confirms the presence of a small amount (<2% at.) of Al as shown by the spectrum in Figure 3.

### 3.3. XPS Analysis 

**Figure 4:**
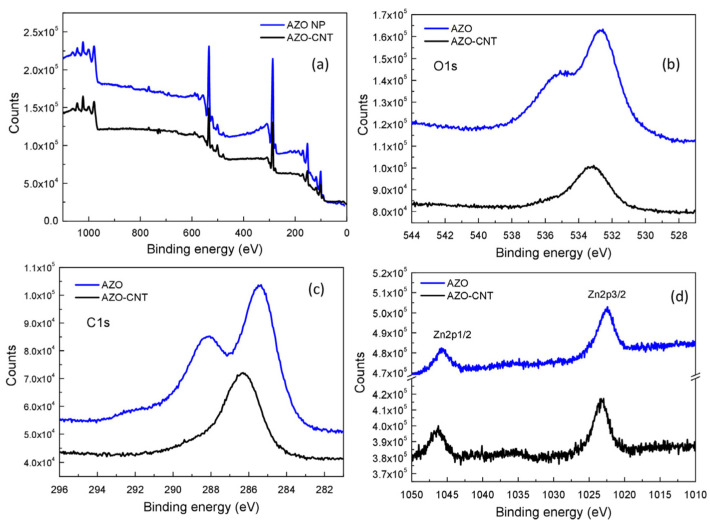
XPS overview spectra (**a**) and (**b**) C1s, (**c**) O1s, and (**d**) Zn2p spectra of AZO–NPs and AZO-decorated CNTs.

The AZO–NPs, CNTs, and AZO–CNT (weight ratio of 4.2) solutions were dropped on silicon substrates and, after drying, they were analyzed by XPS, as reported in Figure 4. In Figure 4a–d we report, respectively, the overview spectrum and the C1s, O1s, and Zn2p spectra for AZO–NPs and for the AZO–CNT samples. The Al2p peak was not observed, since it was below the sensitivity due to the small amount of AZO particles deposited on the silicon substrate and the very low Al concentration contained in the ZnO nanoparticles, as also confirmed by EDX results during TEM analysis. 

Evident double peaks are present in both the C1s and O1s spectra of AZO–NPs, while a single large peak is present in the spectra of AZO–CNT composites (Figure 4b,c,e). Zn2p peaks (Figure 4d) do not show evident differences in shape but a small shift in position for AZO and AZO–CNT samples.

C1s and O1s spectra were deconvoluted to identify different species present on the surface, as shown in Figure S2 of the supplementary materials for AZO and AZO–CNT samples, respectively. The results are summarized in Table 1.

In the case of AZO–NP samples, the C1s peak can be deconvoluted into four peaks (Figure S2a) assigned to carbon atoms coming from contamination, mainly due to alcohol species contained in the original AZO–NPs dispersion. All peaks indicate that C atoms are bound to oxygen as C-O, C=O, O-C=O going from lower (285.4 eV) to higher (289.1 eV) binding energy values. The contribution at 291.5 eV can probably be attributed to residual fragments of alcohol molecules on the AZO surface.

The O1s peak presents four contributions too (Figure S2b): the one at 530.6 eV is known in the literature as due to the O^2-^ state of oxygen in the ZnO lattice [25]; the contributions at 532.4 and 533.6 eV can be associated with O-C and O=C, respectively. Finally, the peak at 535.6 is probably related to the contribution of the C1s peak at 291 eV, i.e., oxygen in a residual alcohol species coming from the original AZO–NP dispersion and adsorbed on the AZO surface.

In the AZO–CNT sample, the C1s peak can be deconvoluted into two peaks (Figure S2c) centered at 286.2 eV and 288.4 eV that can be related to C-O and C=O, respectively. The contribution at 291.5 eV, present in the AZO C1s spectrum, is not visible in the AZO–CNT spectrum anymore, indicating that in the composite material the C and O signals are mainly coming from carboxylic functionalization of CNTs coordinated to Zn atoms. In addition, the C=O/C–O bonds ratio, obtained by ~288 eV and ~286 eV peak areas ratio, is 0.54 for AZO–NPs and 0.24 for the AZO–CNT composite. Even if in the composite a contribution of carboxyl functionalization of CNTs is present, the amount of C-O bonds is greater than in AZO–NPs (where C is present as impurities). The coordination of AZO–NPs with CNTs could occur by coordination of Zn atoms with COOH groups on CNTs’ surface, destabilizing the C=O bond that is converted into C-O (see Figure S3 of Supplementary Materials). 

As far as the O1s peak (Figure S2d) is concerned, it is mainly formed by a large contribution at 533.2 eV due to O-C=O, i.e., the carboxylic groups where Zn atoms coordinate with a CNT, and a very small feature at 535.7 eV related to a reduced amount of impurities due to alcohol species. The peak at 530.6 eV, observed in the AZO sample and related to the O^2−^ in the ZnO lattice, is not present here, suggesting the occurrence of an interaction with CNTs [26].

The C1s:O1s area ratio is 1 for AZO and 5 for AZO–CNT, as expected, since for the composite the higher C1s signal is mainly due to the presence of CNTs.

### 3.4. Electro-Optical Characterization 

**Figure 5:**
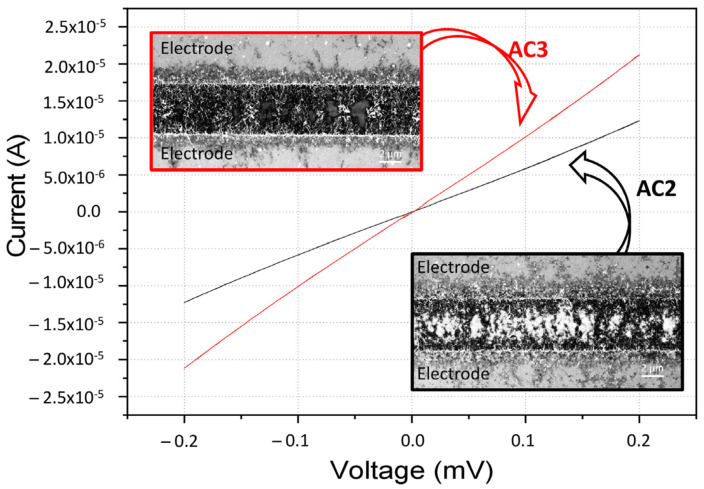
IV measurements performed on two samples with the same AZO:CNT weight ratio (4.2) and two different density layers, lower for AC2 and higher for AC3. SEM images of the active region for the two samples are reported in the insets.

The AZO–CNT layer deposited between two electrodes, as described in the Section 2, was characterized from the electrical point of view. IV measurements were performed in a range between [−0.2 V, +0.2 V], showing an ohmic behavior. In particular, in Figure 5 we report IV measurements and SEM analysis for two different samples (named AC2 and AC3) with the same AZO:CNT weight ratio (4.2) and different layer densities obtained using two different deposition times (5 and 10 minutes, respectively). As expected, the higher density layer (AC3) showed higher current values with respect to the lower density layer (AC2), as reported in Figure 5.

Afterwards, we chose the more conductive sample (AC3) and applied a 2 mV voltage between the electrodes. The sample was mounted vertically in front of a UV lamp, a shutter was placed between lamp and sample, and the current was measured in dark conditions (with shutter) and under UV illumination (no shutter). We determined the response of our AC3 sample under UV light irradiation (200–400 nm wavelength range), with an irradiance value of 1.6 mW/cm^2^; moving from light to dark conditions we measured the current modification (in air) as a function of time. As the shutter is removed, passing from dark to illumination, the current increases very fast and, vice versa, interposing again the shutter, the current decreases back. 

**Figure 6:**
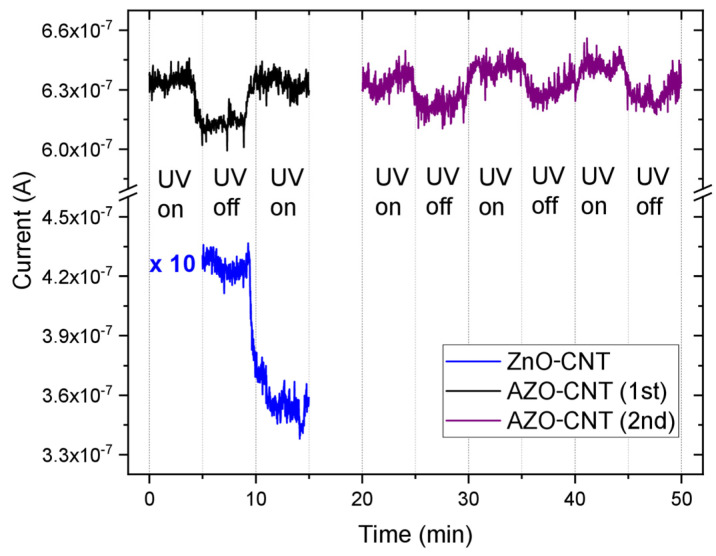
Current flowing at 2 mV through an AZO–CNT layer (AZO:CNT weight ratio = 4.2) during UV light irradiation cycles as a function of time. 1st refers to one cycle, while 2nd refers to other consecutive UVon/UVoff cycles. For comparison, the current flowing at 3 mV in a similar sample containing ZnO-decorated CNTs (ZnO:CNT weight ratio = 11) is also reported. The current values reported for ZnO:CNT are multiplied by a factor of 10 to compare them in the same graph with the values obtained for AZO–CNT layers.

This cycle was repeated several times, and the results can be summarized in Figure 6. 

Figure 6 reports the current change measured at 2 mV through an AZO–CNT layer (AZO:CNT weight ratio = 4.2) during UV light irradiation as a function of time. The black curve shows only one cycle: under irradiation, the current increased and returned to its initial value as the UV light was switched off. The violet curve represents consecutive UVon/UVoff cycles for the same device under the same conditions: the electrical response is repeatable. The blue curve is added for comparison with a similar sample containing ZnO-decorated CNTs (ZnO:CNT weight ratio = 11; operation voltage = 3 mV). This curve showed that for those experimental conditions, the current measured for ZnO/CNT samples is lower than the one measured for the AZO–CNT composite. Furthermore, the electrical response of the two composites is completely different during the UVon/UVoff cycle. The mechanisms related to the electrical response of the AZO–CNT layer under UV irradiation and the comparison with ZnO/CNTs is reported in the following paragraph. 

Before starting the measurement, the device was kept for 1 h under UV light for current stabilization (not shown in Figure 6). In air, oxygen can be adsorbed on both ZnO and AZO surfaces: electrons, photogenerated or due to Al doping, capture the O_2_ molecules and adsorb them as O_2_^-^ on the nanoparticle surface. The binding of free electrons and O_2_ molecules reduces the electrical conductivity. On ZnO, this process is favored by UV irradiation, due to the photoinduced charge, while in AZO it is already possible without illumination, due to the presence of electrons coming from Al doping.

For the AZO compound, under irradiation we observed a clear and sharp increase of the current; then it decreased, turning back to the initial value when the UV light was turned off. The response of the sensor to UV light is exactly the same for many UVon/ UVoff cycles. On the contrary, the current decreased under irradiation for a similar sample where CNTs were decorated by ZnO nanoparticles instead of AZO–NPs [19] (reported in Figure 6 as comparison).

On a clean metal oxide surface, different reactions can occur in the dark or under irradiation in air, resulting in a current change. 

UV irradiation generates excited holes and electrons in the metal oxide (MOx + hv → h^+^ + e^−^). The photoexcited species could recombine without any effect on measured current or be involved in other reaction paths. Electrons could capture oxygen (O_2_ + e^−^ → O_2_^−^), determining a current decrease, or could be transferred directly to MWCNTs, inducing a current increase. Photogenerated holes can recombine with electrons in the MO_X_ nanoparticle or recombine with electrons from MWCNTs. 

**Figure 7:**
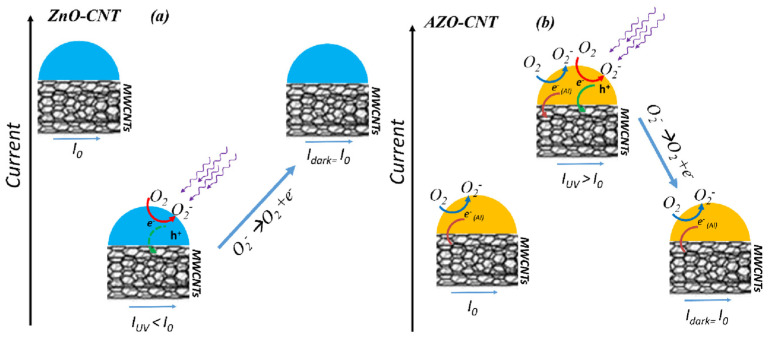
Scheme of involved mechanisms in the dark or under irradiation conditions for both ZnO–CNT (**a**) and AZO–CNT (**b**) composites. In this scheme, we are considering a voltage applied to the composite layer generating a current I_0_ through the MWCNTs. I_UV_ is the current under irradiation, while I_dark_ is the current in dark conditions (I_dark_ = I_0_).

In Figure 7, we report a scheme of all the possible involved mechanisms in dark or under irradiation conditions for both ZnO:CNT (a) and AZO–CNT (b) composites. If a voltage is applied to the composite layer, a current I_0_ flows through the MWCNTs. Under UV irradiation, the current changes (I_UV_), and then turns back to the initial value when the irradiation is switched off (I_dark_ = I_0_).

For ZnO–CNTs, the measured current under UV light decreased, and this behavior is explained as an O_2_ adsorption during irradiation (red arrow in the scheme), with electron transfer from CNT to the metal oxide nanoparticle [19]. Thus, in this case, the oxygen adsorption seems to be favored with respect to electron migration into MWCNT (green arrow in the scheme). Furthermore, photogenerated holes could recombine with electrons in the MWCNTs, resulting in a current decrease (not shown in the scheme). 

To explain the electrical behavior of AZO–NP-decorated CNTs with or without UV light irradiation, besides the O_2_ adsorption/desorption phenomena, we have to consider the presence of electrons introduced by the n-doping of ZnO due to Al. Indeed, in Al-doped ZnO, Al^3+^ substitutes into host Zn^2+^ sites and provides an extra electron (reported as e^−^_(Al)_ in the Figure 7), which increases the carrier concentration density with respect to undoped ZnO [27]. Under UV light exposure, a further increase of carrier concentration is induced on the metal oxide nanoparticle, and when the AZO surface is saturated by adsorbed O_2_ molecules (red arrow in the scheme), the extra charge is transferred to CNTs, producing a current raise (green arrow in the scheme). 

When the light is off, no further photogenerated species are present, and the system returns to its initial current value (i.e., increase for ZnO–CNTs and decrease for AZO–CNTs). 

Using the measurements reported in Figure 7, the current was finally converted to resistance values, and the UV response has been calculated as the modulus of the difference between the resistances measured at UVon and UVoff conditions, normalized to the resistance at UVoff condition, i.e., |R_UVon_ − R_UVoff_|/R_UVoff_. Our results showed a UV response of about 3.6% that is comparable (as absolute value) with the ones obtained for ZnO-CNT based devices where similar electrode sizes and geometry were used [19]. 

## 4. Conclusions

AZO–NP-decorated CNTs were prepared by a very simple, room temperature methodology. As expected, the AZO decoration uniformity depends on the AZO:CNT weight ratio. The AZO-–NPs possess a flattened disk shape, and the contact between AZO NPs and CNTs occurs mainly through the disk face, that is, a (001) plane. Zn atoms of AZO–NPs coordinate with COOH groups on the CNTs’ surface, destabilizing the C=O bond that is converted into C-O, as confirmed by XPS analysis.

AZO–CNT layers, deposited by dielectrophoresis between two electrodes, showed a fast and repeatable electrical response to UV light irradiation: the resistance decreased as the UV light was switched on and rapidly increased as the UV light is switched off. This behavior is the opposite of what occurs in the case of ZnO:CNT layers [19], and it can be explained as follows: in the case of ZnO:CNT, the current decreases under UV irradiation due to O_2_ adsorption and electrons are transferred from CNT to the ZnO nanoparticles, while the O_2_ desorption from the ZnO surface induces electrons to move back to the CNT, determining a current increase. In the case of AZO–NP-decorated CNTs, the extra electrons provided by Al doping to ZnO can favor O_2_ adsorption even in dark conditions. As a voltage is applied to the AZO–CNT layer, an equilibrium condition is reached between the current flowing through the CNTs and the charge (electrons) employed for the adsorption of O_2_ molecules. Under UV light exposure, a further increase of carrier concentration is induced on the metal oxide nanoparticle and, if the AZO surface is already saturated by O_2_ molecules, the extra charge is transferred to CNTs, producing a current raise. The UV light response and sensitivity shown by the AZO–CNT nanocomposite layers contained in a very small area (4 µm × 500 µm instead of typical values between 0.1–1 cm^2^) and operating at low voltage (2 mV instead of typical 10 V), suggest that it is a promising material for UV light sensing applications, with the great advantage that it can operate in air, at room temperature, and at low voltage. The proposed methodology can be extended to low-cost substrates (flexible, plastic substrates) since it does not require high-temperature processes for sensor preparation nor high-temperature working conditions. Furthermore, its response can be proportionally increased just by enlarging the surface area of the sensitive region between the electrodes or using an array of detectors. Further experiments will be performed to investigate the behavior of the sensing layers in other environments, such as in an inert environment or in vacuum, but we expect that the absence of oxygen will affect the AZO–CNT response to UV irradiation less than the ZnO:CNT one, according to the mechanisms indicated in the scheme of Figure 7.

## Figures and Tables

**Table 1:** Results of the XPS peaks deconvolution for AZO–NPs and AZO–CNT samples (from Figure S2).

Peak	AZO			AZO–CNTs		
	Binding Energy (eV)	Area	FWHM	Binding Energy (eV)	Area	FWHM
C1s	285.4	118,003	2.1	286.2	68,669	2.1
	288.0	63,683	2.3	288.4	17,029	2.5
	289.1	22,509	2.5			
	291.5	10,622	2.5			
O1s	530.6	7293	1.7			
	532.4	92,405	2.0	533.2	55,074	2.5
	533.6	50,117	2.5			
	535.6	62,010	2.5	535.7	5027	1.7
Zn2p	1022.7	81,061	2.7	1023.3	119,542	2.6

## Data Availability

Not applicable.

## Supplementary Materials

The following are available online at https://www.mdpi.com/article/10.3390/nano13010215/s1, Figure S1: Spectrum of the light source (ASBN-D130 deuterium light source by Spectral Products (Putnam, CT, USA); Figure S2: Deconvolution of C1s and O1s XPS peaks for AZO–NP samples (a,b) and for AZO–CNT samples (c,d); Figure S3: Scheme of ZnO coordination on carboxylic functionalization on CNTs.
