# Supplementary material for: AZO Nanoparticles-Decorated CNTs for UV Light Sensing: A Structural, Chemical, and Electro-Optical Investigation
Source: Nanomaterials (Basel). 2023 Jan 3;13(1):215. doi: 10.3390/nano13010215 (PMC9823749; doi:10.3390/nano13010215)
Supplement: Supplementary file 1 [file nanomaterials-13-00215-s001.zip › nanomaterials-2120137-supplementary.pdf]

Supplementary Materials

AZO Nanoparticles-Decorated CNTs for UV Light Sensing: A Structural, Chemical, and Electro-Optical Investigation

Simona Filice [1,†], Stefano Boscarino [2,3,*,†], Mario Scuderi [1], Sebania Libertino [1], Clelia Galati [4], Antonio Terrasi [2,3] and Silvia Scalese [1,*]

[1] Consiglio Nazionale delle Ricerche—Istituto per la Microelettronica e Microsistemi (CNR-IMM), Ottava Strada n.5, I-95121 Catania, Italy
[2] Dipartimento di Fisica e Astronomia, Università degli Studi di Catania, via S. Sofia 64, I-95123 Catania, Italy
[3] Consiglio Nazionale delle Ricerche—Istituto per la Microelettronica e Microsistemi (CNR-IMM), sede di Catania—Università, via S. Sofia 64, I-95123 Catania, Italy
[4] STMicroelectronics, Stradale Primosole 5, I-95121 Catania, Italy
* Correspondence: stefano.boscarino@dfa.unict.it (S.B.); silvia.scalese@imm.cnr.it (S.S.)
† These authors contributed equally to this work.

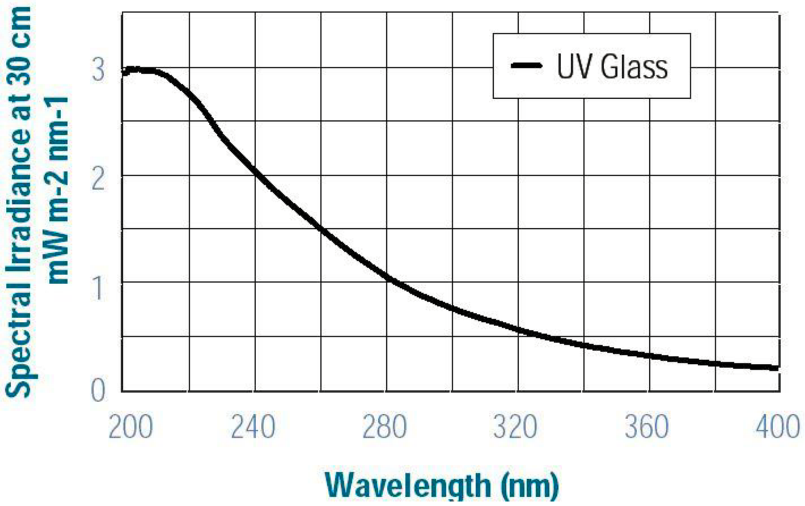

Figure S1: Spectrum of the light source (ASBN-D130 Deuterium Light Source by Spectral Products (Putnam, CT, USA).

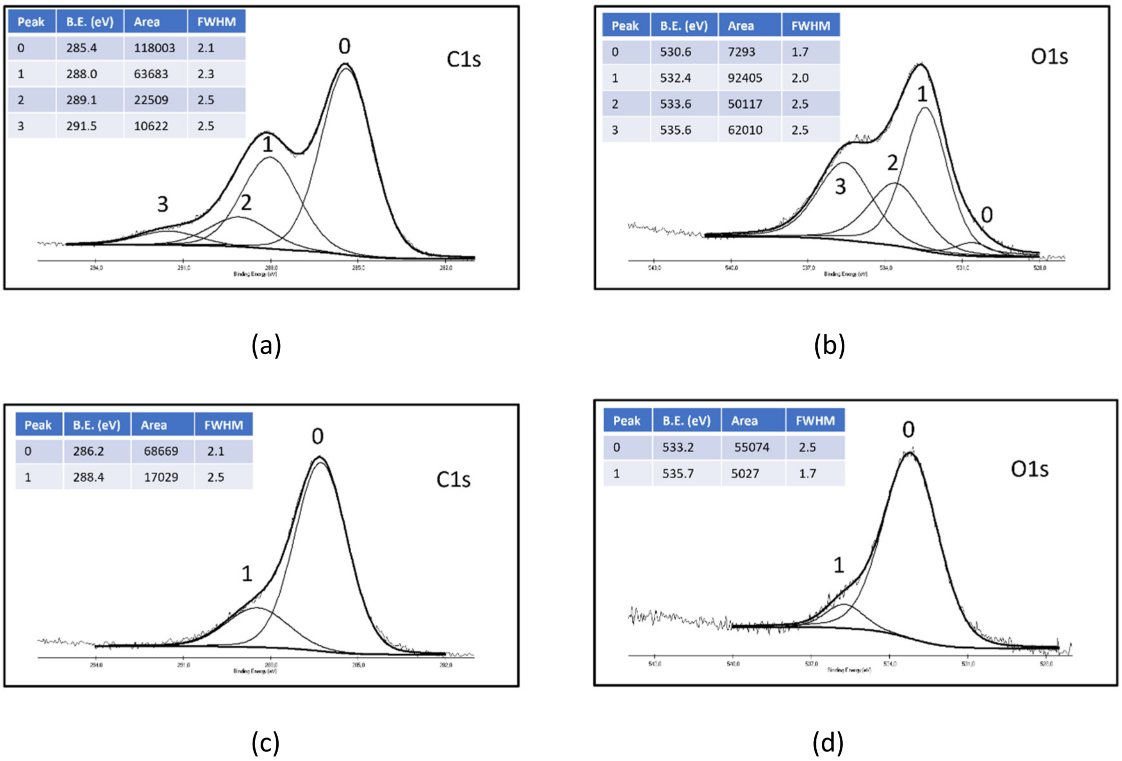

Figure S2: Deconvolution of C1s, O1s XPS peaks for AZO-NPs sample (a, b) and for AZO-CNTs sample (c, d).

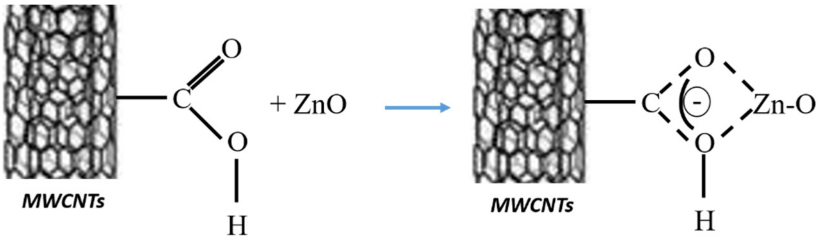

Figure S3: Scheme of ZnO coordination on carboxylic functionalization on CNTs.
